# 2475 The leveling of clinical research competencies

**DOI:** 10.1017/cts.2018.229

**Published:** 2018-11-21

**Authors:** Carolynn T. Jones, Rebecca N. Brouwer, Carmen E. Aldinger, Robert Kolb, William Gluck, Barbara Bierer, Stephen A. Sonstein

**Affiliations:** 1 The Ohio State University; 2 MRCT Center of Brigham and Women’s Hospital and Harvard; 3 Durham Technical Community College

## Abstract

OBJECTIVES/SPECIFIC AIMS: Objectives/goals: Describe the process used to develop leveled competencies and associated examples. Discuss the final leveled competencies and their potential use in clinical research professional workforce initiatives. METHODS/STUDY POPULATION: The revised JTFCTC Framework 2.0 has 51 competency statements, representing 8 domains. Each competency statement has now been refined to delineate fundamental, skilled or advanced levels of knowledge and capability. Typically, the fundamental level describes the competency for a professional that requires some coaching and oversight, but is able to understand and identify basic concepts. The skilled level of the competency reflects the professional’s solid understanding of the competency and use of the information to take action independently in most situations. The advanced level embodies high level thinking, problem solving, and the ability to guide others in the competency. The process for developing both the three levels and examples involved 5 workgroups, each chaired by a content expert and comprising of national/international clinical research experts, including representatives from research sites, professional associations, government, and industry and academic sponsors. RESULTS/ANTICIPATED RESULTS: The committee developed 51 specific competencies arrayed across 3 levels and examples of each to demonstrate an appropriate application of the competency. The competencies and examples, and potential utilization, will be described. DISCUSSION/SIGNIFICANCE OF IMPACT: The use of competencies in the context of workforce development and training initiatives is helping to create standards for the clinical research profession. These leveled competencies allow for an important refinement to the standards that can be used to enhance the quality and safety of the clinical research enterprise and guide workforce development.

